# Discordance Between Ultrasonographic and Clinical Outcomes After Corticosteroid Injection in Sjögren’s Disease-Associated Carpal Tunnel Syndrome

**DOI:** 10.3390/ijms27167266

**Published:** 2026-08-14

**Authors:** Iga Kościńska-Shukla, Arkadiusz Szarmach, Magdalena Chylińska, Dawid Jaskólski, Michał Chmielewski, Natalia Dułak, Magdalena Rytlewska, Michał Olech, Zofia Mikołajczak, Aleksandra Pociej, Marta Jaskólska

**Affiliations:** 1 Department of Rheumatology, Clinical Immunology, Geriatrics and Internal Medicine, Medical University of Gdańsk, ul. Mariana Smoluchowskiego 17, 80-214 Gdańsk, Polandmarta.jaskolska@gumed.edu.pl (M.J.); 2 University Clinical Centre, Gdańsk, ul. Dębinki 7, 80-952 Gdańsk, Poland; arkadiusz.szarmach@gumed.edu.pl (A.S.);; 3 2nd Division of Radiology, Medical University of Gdańsk, ul. Mariana Smoluchowskiego 17, 80-214 Gdańsk, Poland; 4 Division of Adult Neurology, Medical University of Gdańsk, ul. Mariana Smoluchowskiego 17, 80-214 Gdańsk, Poland; 5 2nd Division of Orthopedics and Kinetic Organ Traumatology, Medical University of Gdańsk, ul. Mariana Smoluchowskiego 17, 80-214 Gdańsk, Poland; 6 Division of Quality of Life Research, Medical University of Gdańsk, ul. Tuwima 15, 80-210 Gdańsk, Poland

**Keywords:** Sjögren’s disease, carpal tunnel syndrome, peripheral neuropathy, corticosteroid injection, ultrasonography, median nerve, chronic pain, patient-reported outcomes

## Abstract

Peripheral neuropathy constitutes a prevalent, albeit underrecognized, extraglandular manifestation of Sjögren’s disease (SjD). Carpal tunnel syndrome (CTS) commonly coexists with SjD; however, the efficacy of standard CTS treatments in this population remains insufficiently characterized. This study evaluated the clinical and biological response to local corticosteroid injection in CTS patients with and without SjD and explored factors potentially associated with treatment outcomes. Twenty patients with SjD and CTS diagnosed based on typical clinical symptoms and supportive ultrasonographic findings and 19 control subjects with idiopathic CTS underwent ultrasound-guided corticosteroid injection. Median nerve cross-sectional area (CSA), the Boston Carpal Tunnel Questionnaire (BCTQ), and the Disabilities of the Arm, Shoulder and Hand (DASH) questionnaire were assessed before treatment and 4 weeks after injection. Patients with SjD additionally underwent comprehensive clinical, laboratory, neurophysiological, and patient-reported outcome assessments. Patients with SjD appeared to demonstrate a significantly greater reduction in median nerve CSA following corticosteroid injection compared with controls (mean reduction: 0.46 vs. 0.31 mm^2^; *p* = 0.006, Cohen’s *d* = 0.96, CI90% [0.51, 1.49]). Despite this favorable biological response, clinical improvement was significantly smaller in the SjD group. Patients without SjD exhibited greater improvement in both the BCTQ Symptom Severity Scale (*p* = 0.021) and Functional Status Scale (*p* = 0.033), whereas changes in DASH scores did not differ significantly between groups. Patients with concomitant peripheral neuropathies experienced significantly poorer BCTQ improvement than those with isolated CTS, suggesting that neurological comorbidity may contribute to treatment resistance. No significant associations were identified between treatment response and fibromyalgia status or ESSPRI domains. Among quality-of-life measures, only the SF-36 General Health domain differed significantly between neuropathy types. Patients with SjD and CTS exhibit a dissociation between structural improvement of the median nerve and subjective symptom relief after corticosteroid injection. These findings suggest that mechanisms beyond local nerve compression, including coexisting peripheral neuropathy and potentially altered pain processing, may contribute to persistent symptoms. Comprehensive neurological assessment and multidimensional outcome evaluation may improve identification of SjD patients at risk of suboptimal response to conventional CTS therapies.

## 1. Introduction

Sjögren’s disease (SjD) may manifest as a primary systemic autoimmune disorder or develop secondary to other rheumatic conditions. It is classically characterized by sicca symptoms resulting from lymphocytic infiltration and dysfunction of the exocrine glands [1]. Consequently, patients commonly experience ocular, oral, and vaginal dryness. However, SjD extends far beyond glandular involvement, with manifestations affecting multiple organ systems, including the lungs, kidneys, and both the peripheral and central nervous systems. Peripheral neuropathies are among the most underrecognized extraglandular manifestations of SjD, although their underlying pathophysiological mechanisms appear to be not fully elucidated [2,3,4]. Furthermore, patients with SjD exhibit an increased prevalence of psychiatric comorbidities, particularly depression and anxiety [5], as well as fibromyalgia (FM) [6]. These conditions may amplify pain perception, impair quality of life, and adversely affect the response to both pharmacological and interventional pain management strategies. The epidemiology of SjD remains incompletely defined, with reported prevalence estimates varying widely between studies (0.02–0.77%), largely owing to differences in case ascertainment methods and classification criteria. The true prevalence of SjD may be underestimated because of substantial diagnostic delays and the diverse spectrum of clinical manifestations, especially when extraglandular features precede or overshadow sicca symptoms [7,8,9].

Carpal tunnel syndrome (CTS) is the most common entrapment neuropathy encountered in clinical practice [10] and is frequently observed in patients with rheumatic diseases [11]. While approximately 3–6% of the general population is affected [12], CTS is more commonly found in patients with rheumatic diseases, particularly rheumatoid arthritis (RA), systemic lupus erythematosus (SLE) and systemic sclerosis (SSc) [13,14,15,16]. The assessment of precise prevalence data within these cohorts appears elusive, primarily due to inherent discrepancies in study methodologies and the confounding influence of comorbidities [11]. Typically, the occurrence of entrapment neuropathy in rheumatic diseases is attributed to the articular manifestations, with either active inflammation leading to the narrowing of the carpal tunnel or irreversible deformations of the joints. In addition to splinting and physiotherapy, the administration of local corticosteroid injections around the median nerve is regarded as primary intervention for short-term improvement (for instance while awaiting surgery or before planned physiotherapy), which appears to demonstrate substantial efficacy in reducing symptoms and improving functional capacity [17]. Nevertheless, clinicians managing patients with SjD often observe a less pronounced therapeutic response compared with individuals without autoimmune disease. The mechanisms underlying these differences remain unclear but may be related to the coexistence of systemic inflammation, immunological mechanisms, peripheral neuropathy, chronic pain syndromes, and psychological comorbidities. These observations highlight the need for a better understanding of treatment outcomes in CTS patients with concomitant SjD.

The primary objective of this investigation involves the evaluation of the efficacy of corticosteroid injections in patients with CTS associated with SjD and in patients with idiopathic CTS, with emphasis on both biological and functional outcomes. A structured literature search of MEDLINE, Embase, and Scopus up to July 2026 identified no previous study directly comparing local corticosteroid injection outcomes between SjD-associated and idiopathic CTS. Furthermore, this study sought to identify clinical, laboratory, and disease-related factors associated with a suboptimal therapeutic response and to determine potential predictors of treatment efficacy.

## 2. Results

**Figure 1:**
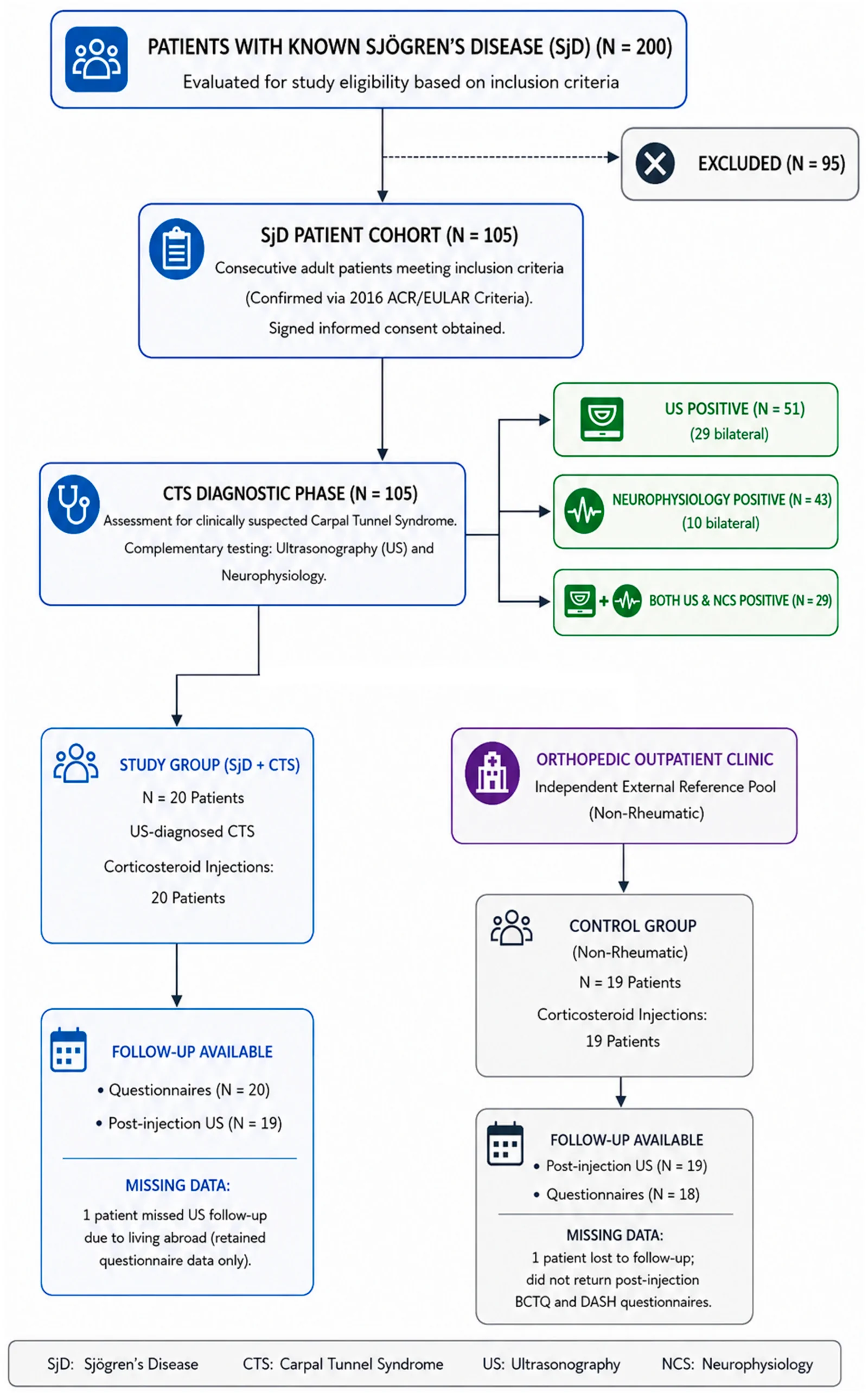
Flowchart of patient inclusion, diagnostic classification, and follow-up.

Among patients with clinically suspected CTS, supportive ultrasonographic findings were observed in 51 patients (29 bilaterally), whereas neurophysiological abnormalities consistent with CTS were identified in 43 patients (10 bilaterally), reflecting the complementary nature and differing diagnostic sensitivity of imaging and electrodiagnostic modalities. Twenty-nine patients presented with features of CTS in both US and neurophysiological testing. All of those diagnosed with CTS were offered therapeutic procedures, but consent was obtained from 20 of them, which aligns with our findings on the patients’ preferences regarding treatment strategies [18]. The study group comprised 20 patients with SjD and CTS diagnosed in the US, while the control group included 19 patients. A total of 35 corticosteroid injections were administered in the study group, with 15 patients receiving bilateral injections. In the control group, 28 injections were performed, including bilateral treatment in 9 patients. One patient from the control group did not return Boston Carpal Tunnel Questionnaire (BCTQ) and Disabilities of the Arm, Shoulder and Hand (DASH) results for post-injection. One patient from the study group did not attend the follow-up visit for the median nerve measurement (the patient lives abroad) and only completed the questionnaires. The complete-case analysis approach was implemented. Interestingly, one of the patients initially screened for the control group with bilateral CTS—upon rheumatological assessment presented symptoms of SjD (dryness of the eyes and oral cavity, peripheral neuropathy). Laboratory testing confirmed the presence of anti-SSA antibodies, and the patient was reclassified to the study group. The flow of patient inclusion is presented in Figure 1.

The mean age was 55.5 ± 12.9 years in the study group and 52.7 ± 13.4 years in the control group. Two participants were men (10.0%) in the study group and 3 participants (15.8%) in the control group. The sex distribution was comparable between groups and reflected the known female predominance of both SjD and CTS. The demographic data are presented in Table 1 and Table 2.

Overall, any form of peripheral nervous system involvement, including isolated CTS, CTS coexisting with other neuropathies, and non-CTS neuropathies, was identified in 69.5% (n = 73) of our cohort, substantially exceeding the prevalence reported in most previous studies and supporting the concept that neurological manifestations of SjD may be underrecognized. The high prevalence of neurological involvement observed in our cohort is consistent with our previous observations indicating that peripheral nervous system manifestations in SjD may be substantially underrecognized in routine clinical practice, particularly when comprehensive neurophysiological and ultrasonographic assessment is performed [19,20]. Among the patients diagnosed in the US with unilateral CTS 15 presented with at least one other form of polyneuropathy (PN) (68%) and among those with bilateral CTS nineteen suffered from other forms of PN (65%).

**Figure 2:**
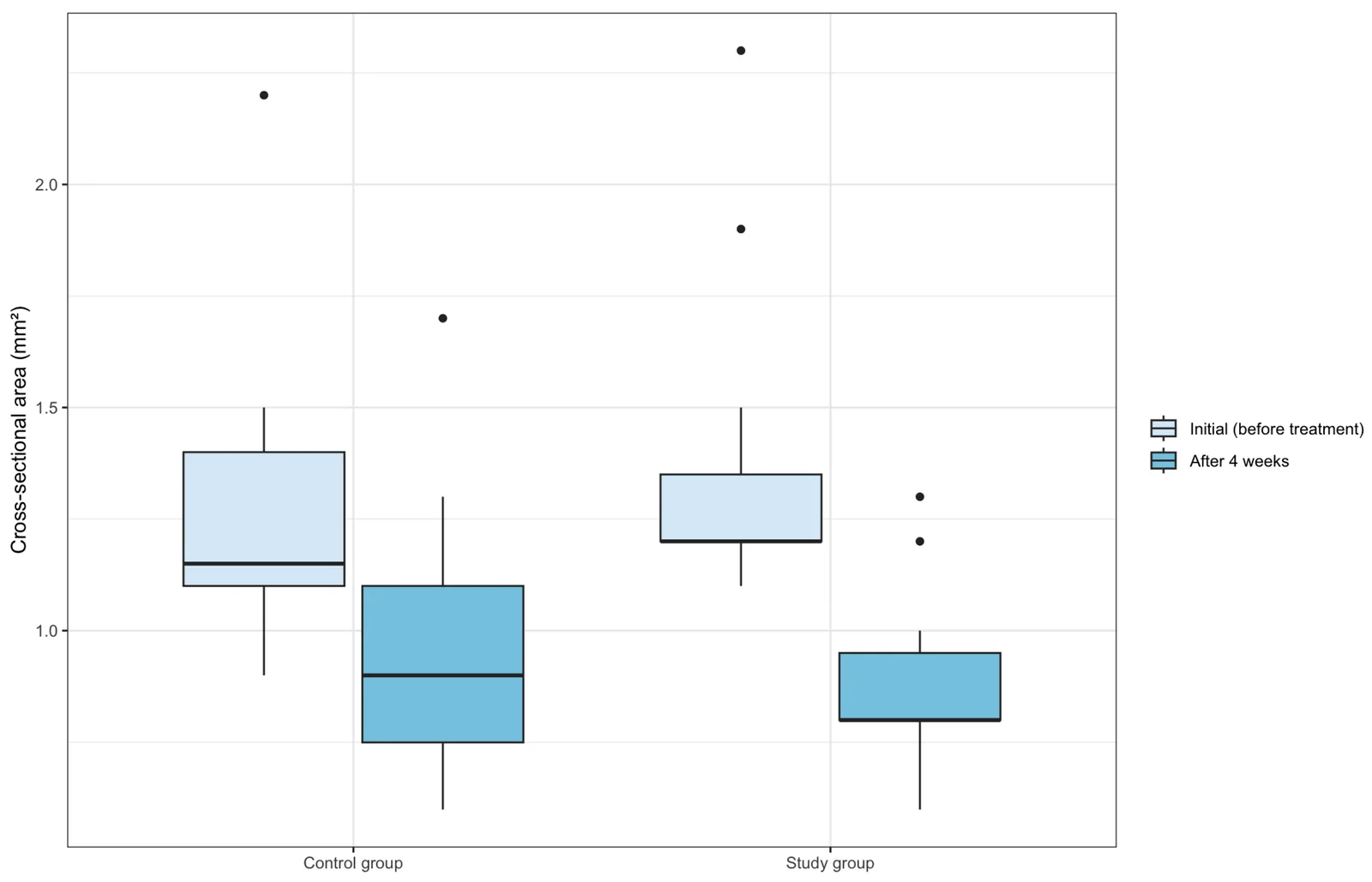
Cross-sectional area of the median nerve over time in the control and study groups.

Comparison of treatment-related changes in median nerve measurements demonstrated a significant difference between patients with SjD and those without the disease. Levene’s test confirmed homogeneity of variances (*p* = 0.163), and an independent-samples *t*-test revealed a significant between-group difference (*t*(36) = −2.95, *p* = 0.006). The effect size was large (Cohen’s *d* = −0.96, CI90% [0.51, 1.49]). Patients with SjD exhibited a mean reduction in median nerve measurement of 0.46 mm^2^, whereas patients without SjD showed a mean reduction of 0.31 mm^2^. Detailed results are presented in Table 3 and Figure 2.

Analysis of the BCTQ demonstrated significant group differences for both dimensions of the instrument. Homogeneity of variances was confirmed for the Symptom Severity Scale (SSS) and Functional Status Scale (FSS). Patients without SjD showed greater improvement in both BCTQ domains compared with patients with SjD. Significant differences were observed for the SSS (*t*(35) = −2.42, *p* = 0.021, Cohen’s *d* = −0.80, CI90% [−1.50, −0.24]) and the FSS (*t*(35) = −2.22, *p* = 0.033, Cohen’s *d* = −0.73, CI90% [−1.31, −0.22]), indicating moderate-to-large effect sizes. In contrast, the difference in DASH score improvement between groups did not reach statistical significance (*t*(35) = −1.90, *p* = 0.066). Results are presented in Table 4.

Subsequent analyses compared BCTQ outcomes between patients with isolated CTS, and patients presenting with both CTS and another neuropathy. Significant differences were identified for both BCTQ dimensions between the subgroups. For the SSS, the comparison yielded *t*(33) = 2.14, *p* = 0.040, with a large effect size (Cohen’s *d* = 0.81, CI90% [0.19, 1.58]). Similarly, the FSS demonstrated a significant difference (*t*(33) = 2.60, *p* = 0.014) and a large effect size (Cohen’s *d* = 0.97, CI90% [0.52, 1.51]). Among the neuropathies CTS was mainly accompanied by mononeuropathy (*p* = 0.023) and axonal sensory-motor polyneuropathy (*p* = 0.043). Axonal-demyelinating polyneuropathy and radiculopathy approached statistical significance (*p* = 0.057 and 0.062 respectively). Details of this analysis are presented in Table 5.

To determine whether patients with CTS exhibited an increased likelihood of comorbid FM, a chi-square test was performed. This assessment, as well as subsequent analyses, was conducted across three cohorts: individuals with CTS, patients with both CTS and another neuropathy, and those presenting with another neuropathy exclusively. The findings suggest an absence of statistical significance (*p* = 0.847, χ^2^ = 1.39, *df* = 4).

A one-way ANOVA performed for the EULAR Sjögren’s Syndrome Patient Reported Index (ESSPRI) domains (dryness, fatigue, pain, joint pain, muscle pain, and neuropathic symptoms) showed no statistically significant differences among the analyzed groups (all *p* > 0.05). The detailed results are presented in Table 6.

Analysis of SF-36 outcomes revealed a group effect on the edge of statistical significance only for the general health domain (*F*(2) = 3.214, *p* = 0.047) before the correction for multiple comparisons. No statistically significant differences were observed for the remaining SF-36 dimensions (presented in Table 7).

Among the hematological parameters, CRP levels differed significantly across groups (*F*(2) = 3.81, *p* = 0.026). Post hoc analysis demonstrated a significant difference in C-reactive protein (CRP) between patients with other neuropathies and those with concomitant CTS and another neuropathy (1.49 ± 2.71 vs. 3.64 ± 5.22 *p* = 0.035, Cohen’s *d* = −0.52, CI90% [−0.88, −0.15]). No significant differences were detected between patients with CTS alone and those with CTS plus another neuropathy (*p* = 0.983), nor between patients with CTS and those with other neuropathies (*p* = 0.073). Nevertheless, in the study group CRP concentrations were within the normal ranges, suggesting that the clinical significance of this finding may be limited. Analysis was also performed for broader inflammatory markers, that is neutrophile to leukocyte rate (N/L), platelet to lymphocyte rate (P/L), and systemic immune inflammation index [(platelets x neutrophiles)/lymphocytes] (SII).

Notably, when comparing two groups of patients: CTS alone vs. CTS and other neuropathy + other neuropathy, a significant difference was noted in the beta2-microglobulin (B2MG) concentrations. However, this analysis is prone to error due to uneven distribution of patients (19 vs. 72 individuals). Nevertheless, consistent with the CRP observations, these values remained within physiological limits, suggesting that their clinical relevance may be limited. Results of laboratory findings analysis are presented in Table 8.

## 3. Discussion

The primary conclusion of this investigation resides in the discrepancy between the biological response of SjD patients to local steroid treatment (understood as decreased median nerve cross-sectional area (CSA)) and a comparatively limited self-reported amelioration in symptoms and function.

The symptoms resulting from CTS are mainly attributed to the compression of an enlarged nerve within the narrow space of the carpal tunnel [10]. This anatomical structure is limited by bones on one side and a thick layer of connective tissue (transverse ligament) on the other side. This mechanism constitutes the rationale for the therapeutic methods currently utilized—steroid injections, laser and extracorporeal shock waves as well as decompression surgeries. Initially, external compression leads to damage in the myelin sheath of the median nerve. If left untreated, the damage progresses into the deeper structures of the nerve, typically culminating in axonal-demyelinating CTS, which manifests as protracted, therapy-resistant complaints.

Steroid injections are considered a primary intervention for short-term relief of symptoms. The exact time span varies largely among the studies, with some reporting improvement as short as 6 weeks, others noting benefit even after 6 months [21,22,23,24]. In clinical practice, a series of 3 repeated injections every 4 weeks are recommended, which provided the rationale for the follow-up period established in this study. The period of 4 weeks is generally considered sufficient to both mitigate the post-injection adverse effects (e.g., transient exacerbation of symptoms and localized pain at the injection site) and sustain the beneficial outcomes of the procedure. The pathomechanism of steroid efficacy is based on three modes of action: anti-inflammatory, antifibrotic, and antiedematous [17]. Lack of initial symptom relief within 4 weeks strongly suggests therapeutic failure, as delayed significant improvement beyond this timeframe is uncommon. Conversely, evaluating outcomes at 1 month captures the peak anti-inflammatory and anti-edematous effects of the steroid, thereby providing indirect insight into the inflammatory component of the underlying pathophysiology. A greater biological response observed in SjD patients may reflect a larger contribution of local inflammatory or edema-related mechanisms within the median nerve. Although the laboratory findings of our participants did not reveal systemically elevated concentrations of inflammatory markers, their accumulation within the nervous tissue may remain plausible and would require histopathological examinations for confirmation.

In prior research, elevated B2MG concentrations were associated with the presence of CTS in patients with SjD [25]. B2MG is regarded as a marker of immune activation and B-cell activity in SjD and has been associated with higher EULAR Sjögren’s Syndrome Disease Activity Index (ESSDAI) scores, autoantibody production, hypergammaglobulinemia and lymphoma risk [26,27]. Although this observation was not replicated in the present cohort, the overall lower B2MG levels may reflect improved disease control due to earlier diagnosis and more targeted treatment strategies. The persistence of symptoms despite low inflammatory marker levels suggests that factors other than ongoing immune activation may contribute substantially to symptom burden and treatment response in SjD.

To date, no consistent association has been established between the clinical severity of carpal tunnel syndrome symptoms and the CSA of the median nerve as measured by ultrasonography [28]. The rationale underlying the anticipated clinical improvement following CSA reduction is rooted in the established pathophysiology of CTS, in which symptoms are principally attributed to elevated intracarpal pressure and the consequent mechanical compression of the median nerve [29,30]. Accordingly, a CSA reduction of 0.46 mm^2^—which, in the majority of cases, restores nerve dimensions to within the normal reference range—would be expected to result in complete symptomatic resolution through alleviation of perineurial oedema and restoration of adequate intraneural blood flow [31,32]. Nevertheless, in a proportion of cases, fibrotic alterations of the median nerve may prevail over the reversible inflammatory component, entailing protracted symptomatology despite demonstrable radiological improvement [33].

Despite a reduction in the CSA of the median nerve, the main mechanism responsible for alleviating the symptoms, our SjD patients reported worse symptomatic and functional results than their otherwise healthy counterparts. It is also noteworthy that 75% of the patients in the study group suffered from CTS bilaterally, as opposed to 47% in the control group. The groups did not differ in terms of professions, with office jobs being the most represented.

Our observation that symptom severity and median-nerve cross-sectional area do not exhibit a consistent cross-sectional association complements the longitudinal findings of Popescu et al. in the post-surgical setting [34]. In their prospective cohort of 100 patients treated with ultrasound-guided 5% dextrose hydrodissection, clinical outcomes and CSA improved in parallel across 6 and 12 months, yet electrodiagnostic recovery was distinctly more modest: only 28.7% of patients achieved improvement of ≥1 Bland category at 6 months despite 56.4% attaining a CSA reduction of ≥2 mm^2^. The authors cautioned against interpreting CSA as a direct linear measure of clinical or electrodiagnostic severity, noting that greater baseline enlargement more likely reflects an underlying structural burden than symptom intensity [34]. Taken together, these data support a reorientation of CSA from a cross-sectional surrogate of symptom severity toward a trajectory marker of disease modification—a distinction that warrants particular consideration in pSjD-CTS, in which inflammatory and fibrotic mechanisms are expected to further uncouple nerve.

The management of patients with SjD is often complicated by the co-occurrence of FM and anxiety-depressive disorders. The prevalence of the former has been reported to range from 20% to 30% [6,35], although clinical observations suggest that this figure may reach 50% [36]. Existing literature suggests that such patients often report inferior self-assessment on the ESSPRI scales and manifest elevated disease activity. However, the pathogenesis of this phenomenon is still unclear. Although the current investigation failed to demonstrate a significant association between the BCTQ results and a concomitant FM diagnosis, the potential influence of this comorbidity cannot be definitely excluded, given the constraints of the participant sample size. Moreover, some of the study participants did not meet the diagnostic criteria for FM solely on the basis of symptom duration and with prolonged observation might require re-evaluation. Routine screening for FM may help identify patients who could benefit from dedicated pain management strategies. FM as well as depressive disorders, when left unmanaged, may lead to the development of central sensitization, an elusive condition, with unsatisfactory treatment strategies. A recent study [37] has demonstrated diminished function of hands in newly diagnosed SjD patients along with higher disease activity and anxiety levels. These findings corroborate established accounts and underscore the imperative for early screening and management of hand dysfunction, alongside comprehensive psychological evaluation in SjD.

Neuropathy is a broadly recognized complication of rheumatic conditions, and in case of diseases such as vasculitis, SLE, SSc, the pathogenesis has been quite well studied and verified. Conversely, the pathogenesis remains poorly elucidated in the context of SjD. Management of SjD-associated neuropathy remains challenging, and therapeutic responses to conventional immunomodulatory treatments are often incomplete [3,38]. Consequently, alternative pain-management strategies, including treatments used for neuropathic pain syndromes such as intravenous lidocaine or ketamine, have attracted increasing interest. However, evidence specific to SjD remains limited [39,40,41,42,43,44].

A plausible interpretation of the observed dissociation between structural and symptomatic improvement is the presence of neuroimmune mechanisms that are not adequately addressed by local corticosteroid therapy. Peripheral nervous system involvement in SjD extends beyond large-fiber neuropathies and frequently includes small-fiber neuropathy (SFN), which may manifest as pain, dysesthesia, burning sensations, and sensory hypersensitivity despite normal conventional neurophysiological findings. Increasing evidence suggests that immune-mediated injury involving B-cell activation, pro-inflammatory cytokines, and autoantibody-associated neuronal dysfunction contributes to the development of neuropathic symptoms in SjD. Consequently, reduction in median nerve swelling following corticosteroid injection may improve the mechanical component of CTS while having limited influence on concurrent neuroimmune processes responsible for pain perception.

In addition, CTS in patients with SjD may occur in the context of a more generalized peripheral neuropathy rather than isolated focal entrapment syndrome. Polyneuropathy is among the most common neurological manifestations of SjD and may affect sensory, motor, or sensorimotor fibers through axonal or demyelinating mechanisms. In such cases, electrophysiological abnormalities and clinical symptoms may reflect diffuse nerve injury extending beyond the carpal tunnel. Structural improvement of the median nerve at the entrapment site may therefore not translate into proportional symptom relief if pathological processes simultaneously affect the nerve proximally or involve additional peripheral nerves. This premise is corroborated by observations including that patients with concomitant neuropathies experienced less favorable clinical outcomes despite demonstrating comparable reductions in median nerve cross-sectional area after treatment.

Although peripheral nervous system involvement is the most frequently recognized neurological manifestation of SjD, central nervous system (CNS) involvement has also been reported and may present with a broad spectrum of clinical manifestations [45,46,47]. The intricate relationship between CNS abnormalities, chronic pain, and central sensitization in SjD remains incompletely elucidated. Future studies integrating neurological, neuroimaging, and pain-processing assessments may help clarify the contribution of central mechanisms to persistent symptoms in this population.

A significant clinical inquiry concerns the identification of patients who may experience persistent symptoms despite objective improvement following CTS treatment. Although ESSPRI is the most used patient-reported outcome measure in SjD, none of its domains differentiated between neuropathy subgroups in our cohort. In contrast, the SF-36 General Health domain was associated with neurological status, suggesting that global health perception may better reflect the multidimensional impact of neuropathic symptoms. While these findings require confirmation in larger cohorts, they indicate that broader quality-of-life measures may be useful in identifying patients at risk of suboptimal treatment response.

The study’s primary strength lies in the multidisciplinary assessment performed by specialists in rheumatology, neurology, radiology, and orthopedics, allowing comprehensive characterization of neurological involvement and exclusion of alternative diagnoses. Factors such as comorbidities and vitamin status were considered; furthermore, systemic treatment regimens remained invariant for all participants throughout the follow-up period, and the recruitment of all participants from a single tertiary university hospital may introduce potential referral bias. For the purpose of this study, data on the small fiber neuropathy was not considered. The relatively modest sample size of the intervention cohort and the abbreviated follow-up period may have limited the ability to evaluate long-term clinical outcomes.

## 4. Materials and Methods

### 4.1. Study Group

A total of 105 consecutive patients with a confirmed diagnosis of SjD were enrolled in the study. Diagnosis was established based on the 2016 ACR/EULAR Classification Criteria [48]. Written informed consent was obtained from all participants prior to inclusion. Each patient underwent a comprehensive clinical, laboratory, and imaging assessment. The evaluation included ultrasonography of the upper limb nerves, neurophysiological examinations, measurement of inflammatory markers (CRP, B2MG, ESR, and fibrinogen), complete blood count, and autoimmune antibody profiling using immunoblot assays.

In addition, participants completed a standardized questionnaire comprising the Athens Insomnia Scale [49], ESSPRI [50], modified to separately assess neuropathic, articular, and muscular pain, the Short Form-36 Health Survey (SF-36) developed at RAND as part of the Medical Outcomes Study [51], and a questionnaire for the diagnosis of FM [52].

None of the patients included in the study group underwent changes in their systemic immunosuppressive treatment during the follow-up period.

The control group consisted of individuals without a previous diagnosis of rheumatic disease who were referred from the orthopedic outpatient clinic for the evaluation and treatment of CTS. All control participants underwent a rheumatological assessment performed by a board-certified rheumatologist and the principal investigator to exclude symptoms or signs suggestive of systemic rheumatic disease. Following clinical and laboratory screening, individuals in whom rheumatic disorders were excluded were included in the control group. For the assessment of treatment outcomes, control participants completed the BCTQ [53] and DASH [54] questionnaire at the same time points as the SjD cohort in relation to corticosteroid injection therapy. However, they did not undergo the extended patient-reported outcome assessment administered to patients with SjD.

### 4.2. Inclusion and Exclusion Criteria

#### 4.2.1. Inclusion Criteria

- Age at least 18 years old;
- Informed consent;
- SjD diagnosis based on the ACR-EULAR 2016 Classification Criteria.

#### 4.2.2. Exclusion Criteria

(1) Age below 18 years old;
(2) Lack of informed consent;
(3) SjD associated with another systemic autoimmune disease;
(4) Other systemic diseases, uncontrolled or in the periods of exacerbation, leading to impaired pain perception such as hypo/hyperthyroidism, diabetes mellitus, dyselectrolytemia, severe vitamin D and B12 deficiencies, anemia, active infections.

### 4.3. Neurophysiological Tests

All participants underwent neurophysiological assessment conducted by an experienced neurologist. Electrophysiological testing consisted of nerve conduction studies (NCS) performed using a Dantec Keypoint G4 EMG/NCS/EP Workstation (Natus) under standardized laboratory conditions, with skin temperatures maintained between 32 °C and 34 °C. All examinations followed the recommendations of the American Association of Neuromuscular and Electrodiagnostic Medicine (AANEM) [55].

Motor nerve conduction studies were carried out on the median, ulnar, radial, and peroneal nerves. Compound muscle action potentials (CMAPs) were recorded from the Abductor Pollicis Brevis, Abductor Digiti Minimi, Extensor Indicis Proprius, and Extensor Digitorum Brevis muscles, respectively.

Sensory nerve conduction studies included evaluation of the median and ulnar nerves using orthodromic techniques, and the radial and sural nerves using antidromic techniques. For the median nerve, sensory nerve action potentials (SNAPs) were elicited by stimulation of digits I and III with recording at the wrist. Ulnar nerve SNAPs were obtained following stimulation of digit V and recording at the wrist. Radial nerve studies involved forearm stimulation with recording at the anatomical snuffbox, whereas sural nerve responses were recorded at the lateral malleolus after stimulation along the lower leg.

A comprehensive overview of the examined nerves and the corresponding laboratory reference values is presented in Table 9. Polyneuropathies were classified according to the criteria proposed by the European Standardized Telematic Tool to Evaluate Electrodiagnostic Methods (ESTEEM) [56], and categorized as axonal, demyelinating, or mixed forms. Electrophysiological abnormalities were further classified into the following diagnostic categories: carpal tunnel syndrome (CTS); mononeuropathy; multiple mononeuropathy (motor, sensory, or sensorimotor); polyneuropathy (demyelinating, axonal sensory, axonal motor, axonal sensorimotor, or mixed axonal–demyelinating); and radiculopathy.

### 4.4. Ultrasonographic Examination

Ultrasound examination of the median nerve was performed using a high-frequency linear-array transducer (≥10 MHz). Patients were examined in a seated position with the forearm supinated, the wrist in a neutral position, and the fingers semi-extended. The median nerve was identified in the transverse plane at the level of the carpal tunnel inlet, corresponding to the pisiform bone and scaphoid tubercle. The CSA of the median nerve was measured by direct tracing of the inner border of the hyperechoic epineurium using the continuous-trace method. Three consecutive measurements were obtained, and the mean value was used for analysis. Care was taken to avoid excessive transducer pressure that could artificially reduce nerve dimensions. In addition to CSA measurement, qualitative assessment of nerve morphology, including nerve swelling, hypoechogenicity, and flattening within the carpal tunnel, was performed when applicable. CTS was diagnosed on the basis of characteristic clinical symptoms together with supportive findings on ultrasonography (a median nerve CSA exceeding 10 mm^2^ at the carpal tunnel inlet), nerve conduction studies, or both. This approach was chosen to reflect routine clinical practice, where CTS diagnosis relies on the integration of clinical presentation and complementary diagnostic modalities rather than on a single test alone.

### 4.5. Intervention

The patients diagnosed with CTS were offered steroid injection. The measurement of the median nerve was confirmed by two independent researchers. Carpal tunnel injections were performed under ultrasound guidance by the same experienced physician. Procedure was done under aseptic conditions with the patient in a supine position and the wrist slightly extended. After skin disinfection, a corticosteroid (7 mg of betamethasone) preparation, without a local anesthetic according to institutional practice, was injected into the carpal tunnel adjacent to the median nerve. The needle was introduced at the level of the proximal wrist crease, typically just ulnar to the palmaris longus tendon (or along an alternative anatomical landmark in its absence), and advanced into the carpal tunnel while avoiding direct contact with the median nerve and surrounding vascular structures. Following a negative aspiration, the injectate was administered slowly. Patients were observed for immediate complications and advised to limit strenuous hand activities for the subsequent 24 to 48 h.

At baseline, prior to corticosteroid injection, patients completed the BCTQ and DASH questionnaires to assess CTS-related symptom severity and upper-extremity disability. BCTQ scores were analyzed separately for the Symptom Severity Scale (SSS) and the Functional Status Scale (FSS).

Symptom severity and functional impairment related to CTS were assessed using the BCTQ, a validated disease-specific patient-reported outcome measure. The BCTQ consists of two subscales: the SSS, comprising 11 items evaluating the intensity and frequency of symptoms, and the FSS, comprising 8 items assessing difficulties in performing daily activities. Each item is scored on a 5-point Likert scale, with higher scores indicating greater symptom burden and functional disability. Mean scores for each subscale were calculated according to the original scoring guidelines. Upper-extremity disability and functional limitations were additionally evaluated using the DASH questionnaire. The DASH is a validated 30-item self-administered instrument designed to assess physical function and symptoms associated with musculoskeletal disorders of the upper limb. Scores range from 0 to 100, with higher scores reflecting greater disability and poorer upper-extremity function.

A follow-up examination was scheduled four weeks following the corticosteroid injections, given the literature-derived consensus regarding the peak efficacy interval for CTS management. Upon the visit, a total of six measurements of the median nerve were performed by two independent researchers. Moreover, the patients were asked to complete the same set of files, including BCTQ and DASH scales.

Biological effectiveness of injection was measured as a difference between the CSA of the median nerve after and before injection. For both instruments, assessments were performed before the intervention and during follow-up after corticosteroid injection. Changes in questionnaire scores were calculated as the difference between post-intervention and baseline values and were used for subsequent statistical analyses.

### 4.6. Statistical Analysis

Statistical analyses were performed using the R programming language and the RStudio 2024.09.0+375 (2024.09.0+375) integrated development environment. All data analyses were conducted in R using several packages (including their dependencies), namely psych 2.4.3 [57], lavaan 0.6-17 [58], and tidyverse 2.0.0 [59].

Statistical analyses were performed to compare treatment-related changes between study groups and to evaluate differences in clinical, patient-reported, and laboratory outcomes. For continuous variables, change scores were calculated as the difference between post-injection and pre-injection measurements. Prior to group comparisons, homogeneity of variances was assessed using Levene’s test. When the assumption of equal variances was met, independent-samples Student’s *t*-tests were used to compare mean changes between groups. Effect sizes were estimated using Cohen’s *d* and interpreted according to conventional thresholds.

Changes in median nerve cross-sectional measurements, BCTQ SSS and FSS scores, as well as DASH scores were analyzed using independent-samples *t*-tests. Additional comparisons of BCTQ outcomes were performed between patients with CTS, patients with other neuropathies, and patients presenting with both CTS and another neuropathy. Because BCTQ and DASH data were available only for a subset of participants, analyses were conducted on complete cases. Because all participants received an injection in the right wrist while only a minority underwent bilateral procedures, statistical evaluations were strictly conducted on the right wrist to ensure a consistent patient-level unit of analysis.

To assess differences in ESSPRI domains (dryness, fatigue, pain, joint pain, muscle pain, and neuropathic symptoms), Short Form-36 (SF-36) dimensions, and hematological laboratory parameters among the three clinical groups, one-way analysis of variance (ANOVA) was applied. For variables demonstrating significant overall group effects, post hoc pairwise comparisons were performed to identify specific between-group differences. Statistical significance was defined as a two-sided *p* value < 0.05.

### 4.7. Ethics Approval and Consent to Participate

The study was conducted in accordance with the principles of the Declaration of Helsinki and approved by the Bioethics Committee of Medical University of Gdańsk (approval no. KB/438/2024, approved on 13 September 2024). Written informed consent was obtained from all participants prior to enrollment in the study.

## 5. Conclusions

Local corticosteroid injection appears to constitute an effective intervention for the attenuation of median nerve swelling in patients with CTS associated with SjD. However, despite the demonstration of a greater biological response—as reflected by a more substantial reduction in median nerve CSA—patients with SjD experienced significantly less amelioration in CTS-related symptomatology and hand function than individuals with idiopathic CTS. This discrepancy suggests that the symptom burden in SjD may not be explained exclusively by local nerve compression. Concomitant peripheral neuropathies, chronic pain mechanisms, central sensitization, and disease-related alterations in pain perception may substantially influence therapeutic outcomes. From a clinical perspective, structural improvement as documented via imaging may not necessarily constitute a reliable surrogate for patient-perceived benefit in SjD. Routine screening for PN, FM, and psychological comorbidities may facilitate a more individualized therapeutic approach and help identify patients at risk of persistent symptoms despite a technically successful intervention. The present findings suggest that CTS in SjD may represent a multifactorial disorder in which mechanical nerve compression interacts with systemic neuroimmune mechanisms. Larger prospective studies incorporating neurophysiological, immunological, and molecular biomarkers are warranted to clarify the pathways responsible for the observed dissociation between biological and clinical treatment responses and to identify predictors of therapeutic efficacy.

## Figures and Tables

**Table 1:** Age distribution of the study sample.

Group	*M*	*SD*	Min	Max
Database	55.56	13.89	23	82
SjD injections	55.47	12.93	37	78
SjD-free injections	52.65	13.41	27	73

**Table 2:** Sex composition of the study sample.

Group/Subgroup		N (%)		
		Database	SjD Injections	SjD-Free Injections
Total		125 (100)	20 (100)	19 (100)
Gender				
	Male	11 (8.8)	2 (10)	3 (15.8)
	Female	114 (91.2)	18 (90)	16 (84.2)

**Table 3:** Descriptive statistics for the difference in median nerve measurements [mm^2^].

Group	*m*	*SD*	*skew*	*n*
Sjögren’s disease	−0.46	0.18	−1.26	19
Without Sjögren’s disease	−0.31	0.13	−0.33	19

**Table 4:** Descriptive statistics for changes in BCTQ dimension scores.

Dimension/Group		*M*	*SD*	*skew*	*n*
BCTQ SSS					
	Sjögren’s disease	−6.13	9.80	−0.82	19
	without Sjögren’s disease	−14.16	10.38	−0.46	18
BCTQ FSS					
	Sjögren’s disease	−1.42	5.86	1.27	19
	without Sjögren’s disease	−6.39	7.66	−0.36	18

**Table 5:** Descriptive statistics for the differences between BCTQ dimension scores.

Dimension/Group		*M*	*SD*	*skew*	*n*
BCTQ SSS					
	CTS	−12.94	10.81	−0.35	24
	CTS + other neuropathy	−4.98	8.7	−1.13	11
BCTQ FSS					
	CTS	−5.95	6.98	−0.45	24
	CTS + other neuropathy	0.43	6.15	1.52	11

**Table 6:** Results of the univariate analysis of variance (ANOVA) for ESSPRI scores.

Dependent Variable	F	df	df	*p*-Value
Dryness	1.36	2	63	0.265
Fatigue	0.83	2	63	0.443
Pain	0.84	2	63	0.436
Joint pain	1.93	2	62	0.154
Muscle pain	1.52	2	62	0.228
Neuropathic	1.01	2	62	0.370

**Table 7:** One-way ANOVA results for SF-36 questionnaire scores.

Dependent Variable	F	df	df	*p*-Value
Vitality	1.75	2	59	0.183
Physical functioning	0.23	2	59	0.793
Bodily pain	1.46	2	59	0.242
General health	3.21	2	59	0.047
Role Limitations due to Physical Health	0.87	2	59	0.426
Role Limitations due to Emotional Problems	2.99	2	59	0.058
Social functioning	0.20	2	59	0.820
Mental health	0.41	2	59	0.665
Total	2.22	2	58	0.117

**Table 8:** Laboratory tests results.

Dependent Variable	CTS			CTS + Other Neuropathy			Other Neuropathy			F	df	df	*p*
	*M*	*SD*	*N*	*M*	*SD*	*N*	*M*	*SD*	*N*				
CRP [mg/L]	1.32	1.57	19	1.49	2.71	44	3.64	5.22	28	3.81	2	88	**0.026**
ESR [mm/h]	13.00	13.10	19	15.98	14.96	44	19.07	16.70	28	0.93	2	88	0.399
FBG [g/L]	3.25	0.58	19	3.42	0.65	44	3.46	0.67	28	0.68	2	87	0.508
Hgb [g/dL]	13.81	1.09	19	13.22	1.28	44	13.31	1.36	28	1.44	2	88	0.243
Fe [ug/dL]	101.79	34.17	19	99.55	33.26	44	88.38	38.20	28	1.14	2	88	0.326
Vit. B12 [pg/mL]	484.89	216.96	19	510.30	255.71	44	441.32	182.48	28	0.79	2	88	0.459
B2MG [mg/L]	1.94	0.52	19	2.17	1.09	44	2.61	1.45	28	2.20	2	88	0.117
N/L	1.65	0.71	19	2.57	2.13	44	2.36	1.23	28	2.04	2	88	0.136
P/L	140.46	48.26	19	191.24	104.35	44	179.67	87.14	28	2.12	2	88	0.126
SII	382.93	167.03	19	630.83	616.66	44	596.10	396.48	28	1.77	2	88	0.177

Abbreviations: CRP—C-reactive protein; ESR—erythrocyte sedimentation rate; FBG—fibrinogen; Hgb—hemoglobin; Fe—iron; vit. B12—vitamin B12; B2MG—beta2-microglobulin; N/L—neutrophiles to leukocytes; P/L—platelets to lymphocytes; SII—systemic immune inflammation index. Statistically significant results marked in bold.

**Table 9:** Characteristics of the nerves included in the electrophysiological assessment and the corresponding laboratory reference ranges established for the SjD study population.

Nerve	Amplitude LLN	Distal Latency ULN [Distance]	Conduction Velocity LLN
Motor nerve conduction study			
Median (APB)	5 mV	4.2 ms [80 mm]	50 m/s
Radial (EIP)	3.5 mV	3.5 ms [120 mm]	51 m/s
Ulnar (ADM)	7 mV	3.2 ms [80 mm]	50 m/s
Peroneal (EDB)	3 mV	4.5 ms [80 mm]	40 m/s
Sensory nerve conduction study orthodromic			
Median dig I	10µV	3.5 ms [80 mm]	49 m/s
Median dig III	10 µV	3.4 ms [80 mm]	50 m/s
Ulnar dig. V	8 µV	3.5 ms [80 mm]	50 m/s
Sensory nerve conduction study antidromic			
Radial	10 µV	3.7 ms [100 mm]	51 m/s
Sural	9 µV	4.5 ms [100 mm]	40 m/s

Abbreviations: LLN, Lower Limit of Normal; ULN, Upper Limit of Normal; APB, Abductor Pollicis Brevis muscle; EIP, Extensor Indicis Proprius muscle, ADM, Abductor Digiti Minimi muscle, EDB, Extensor Digitorum Brevis muscle; dig, digit; mV, millivolt; ms, milliseconds; mm, millimeter; µV, microvolt; m/s, meters per second.

## Data Availability

The original contributions presented in this study are included in the article. Further inquiries can be directed to the corresponding author.
